# A realism-based approach to an ontological representation of symbiotic interactions

**DOI:** 10.1186/s12911-020-01273-0

**Published:** 2020-10-08

**Authors:** Matthew Diller, Evan Johnson, Amanda Hicks, William R. Hogan

**Affiliations:** 1 Department of Health Outcomes and Biomedical Informatics, University of Florida, Gainesville, FL USA; 2 Applied Physics Laboratory, Johns Hopkins University, Baltimore, MD USA

**Keywords:** Ontology, Symbiosis, Realism, Host-pathogen interaction

## Abstract

**Background:**

The symbiotic interactions that occur between humans and organisms in our environment have a tremendous impact on our health. Recently, there has been a surge in interest in understanding the complex relationships between the microbiome and human health and host immunity against microbial pathogens, among other things. To collect and manage data about these interactions and their complexity, scientists will need ontologies that represent symbiotic interactions as they occur in reality.

**Methods:**

We began with two papers that reviewed the usage of ‘symbiosis’ and related terms in the biology and ecology literature and prominent textbooks. We then analyzed several prominent standard terminologies and ontologies that contain representations of symbiotic interactions, to determine if they appropriately defined ‘symbiosis’ and related terms according to current scientific usage as identified by the review papers. In the process, we identified several subtypes of symbiotic interactions, as well as the characteristics that differentiate them, which we used to propose textual and axiomatic definitions for each subtype of interaction. To both illustrate how to use the ontological representations and definitions we created and provide additional quality assurance on key definitions, we carried out a referent tracking analysis and representation of three scenarios involving symbiotic interactions among organisms.

**Results:**

We found one definition of ‘symbiosis’ in an existing ontology that was consistent with the vast preponderance of scientific usage in biology and ecology. However, that ontology changed its definition during the course of our work, and discussions are ongoing. We present a new definition that we have proposed. We also define 34 subtypes of symbiosis. Our referent tracking analysis showed that it is necessary to define symbiotic interactions at the level of the individual, rather than at the species level, due to the complex nature in which organisms can go from participating in one type of symbiosis with one organism to participating in another type of symbiosis with a different organism.

**Conclusion:**

As a result of our efforts here, we have developed a robust representation of symbiotic interactions using a realism-based approach, which fills a gap in existing biomedical ontologies.

## Background

From vector-borne diseases to the gut microbiome, symbiotic interactions between humans and other organisms are known to have an immense impact on our health [1, 2]. Over the past century and a half, our understanding of these interactions has evolved so that we now know that the nature of each interaction is not static, but rather tends to be fluid. For example, a given interaction between a human and a colony of *Staphylococcus aureus* (NCBI taxon identifier [taxid] 1280) may begin as a commensal interaction, but later convert into a parasitic one—an infection.

As scientists and clinicians seek to increasingly understand the timing of these interactions (including when one interaction ends and another one begins, as in the previous example), the type of interaction that occurred, and the outcomes for each participant in the interaction, it will be necessary to collect more data about such interactions and to facilitate data sharing and integration. To accomplish these goals, an essential first step is to develop ontologies that rigorously capture in their representational content the complex nature of symbiotic interactions as they exist on the side of the organisms that participate in them.

Unfortunately, since its introduction into biology, the term ‘symbiosis’ has imposed a significant burden upon scientists striving for terminological consistency. Anton de Bary, who is widely cited as having first introduced the term into scientific usage in 1879, intended ‘symbiosis’ to be a general term that denotes intimate interactions between organisms of different species independently of outcome [3]. Shortly thereafter, biologists began to apply the word liberally both to intimate interactions between two organisms of different species in general and often, more narrowly, to interspecies interactions that are mutualistic in nature [4], which has been the cause of a debate that has persisted for years. Recently, there has been a trend toward a general acceptance of the “de Bary definition” in the scientific literature [5, 6].

In this paper, we compare trends in scientific usage with existing semantic resources (i.e., ontologies, terminologies, thesauri) and how they define ‘symbiosis’ and its associated terms. We then propose textual and axiomatic definitions for ‘symbiosis’ and its associated terms according to the principles of ontological realism as outlined by the Open Biomedical Ontologies (OBO) Foundry [7]. In particular, we define ‘symbiosis’ according to current usage, which is in the same spirit as the “de Bary definition,” maintaining that symbiotic interactions are carried out at the level of the individual organism and that there must exist some degree of intimate association during the interaction. Lastly, we illustrate the usage of our representations of symbiotic interactions by conducting a referent-tracking based analysis and representation of three scenarios—two that are relevant to infectious disease epidemiology and one that is more general to the fields of biology and ecology. Referent tracking is a method for explicitly representing particulars in reality and the relationships that exist between them and the types that they instantiate [8]. Note that in the past, we and others have found referent-tracking based analysis to also provide a rigorous test of ontological definitions [9–11], so our referent-tracking analysis also provides quality assurance of key definitions. We intend our work here to serve as a foundation for future data collection on interspecies interactions both within and outside of biomedicine.

## Methods

Our motivation for representing symbiotic interactions arose out of our work with the Apollo Structured Vocabulary (Apollo-SV)—an application ontology that covers population biology and infectious disease epidemiology [12]—and the need to represent interactions among pathogens, hosts, and vector organisms. Apollo-SV is licensed under a Creative Commons Attribution 4.0 International License and can be found at https://github.com/ApolloDev/apollo-sv. All these interactions occur among organisms of different species and have various beneficial and untoward effects for their various participants. Recalling past controversies over whether the term ‘symbiosis’ refers to all interspecies interactions or merely mutualistic ones, we were concerned with aligning our usage with current, scientific best practice and usage.

Therefore, we looked at work that (a) analyzed and explained recent trends in the usage of ‘symbiosis’ in the fields of biology and ecology, (b) defined some of the basic characteristics of symbiotic interactions, and (c) provided a comprehensive set of terms for the various subtypes of symbiotic interactions [5, 6]. We also reviewed the historical usage of ‘symbiosis’ in biology and ecology, as well as current and historical usage of related terms like ‘mutualism’, ‘commensalism’, and ‘parasitism’. From these resources, we compiled a comprehensive list of terms that describe symbiotic interactions in nature.

Based on our analysis of this literature, we concluded that the broader de Bary definition is the overwhelming consensus scientific usage, both current and historical, and thus it should be our starting point for evaluations of existing terminologies, ontologies, and thesaurii. We then searched for ‘symbiosis’ and related terms from ontologies, terminologies, and thesauri using the Ontobee and BioPortal web services [13, 14]. In particular, we searched for representational units from these artifacts that include in their definition of ‘symbiosis’ that 1) the interaction is a process or, more specifically, that it is a biological process, 2) the interaction occurs between individual organisms of different species, and 3) some degree of intimacy is required for the interaction to occur. When we identified a class for re-use, we imported it into Apollo-SV using the Minimum Information to Reference an External Ontology Term (MIREOT) plugin for the Protégé ontology editor [15]. For terms for which we did not find a corresponding class for re-use in a Web Ontology Language (OWL 2) ontology, we created classes in Apollo-SV according to the methods and principles of ontological realism [16]. For each class that we created, we also created a textual definition and a logical axiom in OWL 2.

### Referent tracking analysis

Just as ontologies offer a way to represent types of things and the relationships that exist among them, referent tracking is a paradigm for explicitly representing individuals, or particulars, in reality. These relationships are stored in referent tracking tuples and are meant to offer an explicit reference to those entities in a domain that are explicitly or implicitly mentioned in a particular data source. An example of an entity that is implicitly mentioned might be the right arm for a patient that has presented to the emergency department of a hospital with a right arm fracture. At present, electronic health record systems would represent this with a diagnostic code in some diagnosis field in the patient’s health record. In the past, referent tracking based analyses have been applied to a broad range of disciplines for various purposes, such as the automation of diagnostic algorithms for schizophrenia [17], managing corporate memories [18], sharing intelligence data [19], command and control messaging systems [20], an automated risk management system for monitoring patient safety [21], and for managing digital rights [22]. Our intention here was to demonstrate the feasibility of extending it to the life sciences—in particular, zoology, mycology, ecology, epidemiology, and microbiology—while also using it as a quality check for our ontology class definitions.

As stated previously, we additionally illustrate the use of our ontological representations and test their level of rigor through a referent tracking analysis. To do so, we first constructed three scenarios that involve various types of symbiotic interactions and that are applicable to various domains of the life sciences. The goal for each was to determine whether our definitions are sufficient to capture all the entities that they are meant to capture: primarily, is each interaction in each scenario an instance of the class, as we have defined it? If not, then our definition(s) require revision to improve their accuracy.

Referent tracking analysis follows three main steps. The first step is to identify all of the particulars that are either explicitly or implicitly referenced by the scenario. Each particular is assigned an IUI of the form ‘IUI-n’, where ‘n’ is any positive integer. The second step is to identify the types that each particular instantiates, as well as all relevant temporal regions, including those in which each particular exists and when various particular–particular and particular–type relations hold. Each temporal region is also assigned an identifier of the form ‘t_n_’. where ‘n’ is any positive integer. The final step involves determining the relationships that each particular has with other particulars. Please note that all of the individuals and the details about them in these examples are fictional.

#### Scenario 1: Trichophyton rubrum in humans (ectoparasitism) (domains: epidemiology and mycology)

Mr. Jones is an amateur practitioner of Brazilian jiu-jitsu who, during a recent training session, came in contact with *Trichophyton rubrum* (taxid 5551). As a result, he now has ringworm on the elbow of his right arm for which he is currently seeking medical care.

#### Scenario 2: *Plasmodium vivax* and *Anopheles gambiae* mosquitoes (obligatory endocommensalism, obligatory ectoparasitism, and obligatory endoparasitism) (domains: epidemiology, microbiology, and entomology)

A mosquito of the species *Anopheles gambiae* (taxid 7165) bites Mr. Joaquin, who is infected with *Plasmodium vivax* (taxid 5855). Many parasites enter the mosquito and eventually reproduce to create thousands of sporozoites, which migrate to the mosquito’s salivary glands. The mosquito then bites Mrs. Chang, an uninfected human, which leads to dozens of sporozoites entering her bloodstream and, ultimately, infecting her (making her a host).

#### Scenario 3: Buchnera aphidicola and *Acyrthosiphon pisum* [pea aphid] (obligatory endomutualism) (domains: ecology, microbiology, and entomology)

A pea aphid of the species *Acyrthosiphon pisum* (taxid 7029) is colonized by an aggregate of *Buchnera aphidicola* (taxid 9), which reside within specialized bacteriocyte cells in the aphid host. In return, each *B. aphidicola* bacterium (taxid 9) produces essential amino acids (e.g., tryptophan), which it provides to its *pea aphid* host for nutrition.

## Results

In total, we ended up importing from existing ontologies three classes relevant to symbiosis that met our criteria. We had to create 35 new classes in Apollo-SV for various subtypes of symbiosis and related phenomena. We created textual definitions for the 35 new classes.

### Review of current ontologies, terminologies, and thesauri that define ‘symbiosis’

In total, our search of Ontobee returned eight results, while our search of BioPortal returned 15 results (as of February 8, 2019). Some of the more prominent resources that have a class or “concept” for ‘symbiosis’ include the Computer Retrieval of Information on Scientific Projects (CRISP) Thesaurus [23], Medical Subject Headings (MeSH) [24], the Systematized Nomenclature of Medicine Clinical Terms (SNOMED CT) [25], and the Gene Ontology (GO) [26, 27]. Unfortunately, three of these four (CRISP, MeSH, and SNOMED CT), at the time of this writing, do not use the same upper level ontology or relations as Apollo-SV, which complicates their re-use. Nevertheless, below we discuss the strengths and/or weaknesses of their definitions for ‘symbiosis’.

#### CRISP thesaurus

The CRISP Thesaurus was developed by the National Institutes of Health (NIH) to support the indexing of information about research projects in the CRISP database, which was later replaced by the Research Portfolio Online Reporting Tools Expenditures and Results (RePORTER) query tool. Although the NIH has not updated the CRISP Thesaurus since 2006, it is still available online to download and view. It contains for each term annotations for definitions, synonyms, and unique identifiers, among other things.

The CRISP definition for *symbiosis* (CSP:1035–7338) is: *“[A] state of close, permanent, or obligatory contact between 2 species, usually of a mutually beneficial nature.”* The first problem with this definition is one of granularity, in that it defines a symbiotic interaction as being between two *species*, as opposed to two *organisms* of different species. When a physician informs a patient that he or she has a *S. aureus* (taxid 1280) infection, the physician does not mean that the patient has been infected by the *S. aureus* species (taxid 1280), or that the *Homo sapiens* species (taxid 9606) has been infected with the *S. aureus* species (taxid 1280). Interactions occur between organisms of differing species, not between the species themselves. The second problem is that the definition is too restrictive, as it asserts that symbiotic interactions are “usually of a mutually beneficial nature,” and therefore does not account for interactions in which at least one of the interacting organisms is harmed (e.g., parasitism). Further evidence of this is apparent in the synonyms of ‘symbiosis’ in CRISP, which are ‘mutualism’, ‘commensalism’, and, oddly, ‘commensal organism’.

#### MeSH

MeSH was created and is currently maintained by the United States National Library of Medicine (NLM). It is used primarily for indexing publications in the MEDLINE/PubMed database. Like the CRISP Thesaurus, MeSH’s term hierarchy is organized in a tree-like structure not organized by **is-a** subsumption relations, with each term being assigned a unique identifier, label (i.e., “MeSH Subject Heading”), definition (i.e., “MeSH Description”), and synonyms and alternative labels.

MeSH’s definition of *Symbiosis* (D013559) also failed to meet our criteria. MeSH defines *Symbiosis* (D013559) as, *“A form of symbiosis between two organisms of different species in which one of them benefits from the association whereas the other is largely unaffected or not significantly harmed or benefiting from the relationship.”* Although MeSH does not make the mistake of defining *Symbiosis* (D013559) at the species level, the critical flaw is that its definition is circular, as it defines *Symbiosis* as “[a] form of symbiosis.” Another mistake that MeSH makes is that, like the CRISP Thesaurus, its definition is too narrow since it does not include interspecies interactions in which one or more organisms are harmed.

#### SNOMED CT

SNOMED CT describes its purpose as representing healthcare terminology, and it is used for encoding information in electronic health records. It contains over 311,000 classes (called “concepts”) that are assigned a unique concept identifier (CID). Although many classes in SNOMED CT are assigned a definition (“description”), there are many that do not have one, such as primitive classes. Unlike the CRISP Thesaurus and MeSH, SNOMED CT also contains formal relations to relate classes to each other.

Because *Symbiosis* (CID:49105004) in SNOMED CT is a primitive class, it does not have a definition in the form of logical axioms. Like the vast majority of SNOMED concepts, it also lacks a textual definition. However, given its place in the hierarchy and the synonyms assigned to it, we were nevertheless able to determine that it did not meet our criteria for re-use. Specifically, the first issue with SNOMED CT’s *Symbiosis* (CID:49105004) class is that it is not considered an interaction, but instead is considered a type of adaptation, given that its parent class is *Adaptation, function (observable entity)* (CID:4452006). We find this classification to be ontologically inaccurate since biologists and ecologists typically do not define symbiotic interactions as being adaptations or functions of individual organisms. The second problem with the class is that the synonyms that are assigned to it include ‘Commensalism’ and ‘Symbiosis, function’, which again are too narrow in scope. As stated previously, restricting symbiotic interactions to be exclusively commensal in nature is inconsistent with scientific usage.

#### GO

The GO is a biomedical ontology that provides standard representations for gene products across multiple organism species. As a member of the OBO Foundry, it is developed and maintained according to the principles and standards of ontological realism. Although the scope of GO is primarily centered on gene products, its biological process namespace does contain classes for interactions between organisms, including *interspecies interaction between organisms* (GO:0044419) and *symbiont process* (GO:0044403).

Unfortunately, we discovered that the definition of *symbiont process* (GO:0044403) in GO is also inadequate for reasons similar to the ones described already. GO defines *symbiont process* as *“A process carried out by symbiont gene products that enables a symbiotic interaction with a host organism.”* In addition to being circular, we found that the use of ‘host organism’ implies a narrower definition of ‘symbiosis’ than what de Bary intended, as it excludes symbiotic interactions that do not involve a host. For example, lichens are a composite of algae or cyanobacteria plus fungi and the fungi are not hosts for the algae or cyanobacteria.

Moreover, we discovered that GO had originally assigned a different term and definition to *symbiont process* (GO:0044403), which we actually found to be more appropriate:

- ‘symbiosis, encompassing mutualism through parasitism’ *= def. - An interaction between two organisms living together in more or less intimate association.*

However, the current release of GO now contains the new, circular definition, as well as two new classes—*interaction with host* (GO:0051701) and *interaction with symbiont* (GO:0051702)—which are both currently defined exactly the same as *“An interaction between two organisms living together in more or less intimate association”* (i.e., the previous definition for *symbiosis, encompassing mutualism through parasitism* (GO:0044403)). For these reasons, our re-use of GO classes was limited to *multi-organism process* (GO:0051704) and *interspecies interaction between organisms* (GO:0044419). We logged issues on the GO tracker highlighting the problems with these definitions. The GO team subsequently changed the definition of *symbiont process* (GO:0044403) to: *“A process carried out by symbiont gene products that enables the interaction between two organisms living together in more or less intimate association.”* Now symbiosis is not the interaction among two or more organisms of different species, but merely cellular processes that “enable” the interaction. This definition remains inadequate to our purposes as it excludes the interaction itself. Discussions with GO are ongoing.

#### IDO

IDO consists of a core ontology that covers infectious diseases and several extension ontologies that focus on various infectious disease subdomains (e.g., HIV, Dengue fever). Like GO, it is a member of the OBO Foundry and therefore is developed and maintained according to the principles and standards of ontological realism. Because symbiotic interactions are a central aspect of the domain of infectious diseases, IDO contains several classes that represent such interactions that it imports from GO (e.g., *symbiosis, encompassing mutualism through parasitism* GO:0044403). It also contains classes for the organisms that participate in them and the roles that are realized (e.g., *host* and *host role*, *symbiont* and *symbiont role*). However, these organism and role classes rely on GO’s *symbiosis, encompassing mutualism through parasitism* class (GO:0044403) and therefore run into the same issues that GO is faced with from its definition of *symbiosis, encompassing mutualism through parasitism* (GO:0044403). As such, we did not re-use these classes.

### Defining ‘symbiosis’ and related terms

Given the ongoing and unresolved inadequacy of current representations of ‘symbiosis’ in other ontologies, terminologies, and thesauri, we propose the following definition and equivalent class axiom:

- *symbiosis = def. - An interspecies interaction between two or more organisms in intimate association.*
- ‘interspecies interaction between organisms’ and ((‘has participant’ min 2 (‘organism’ and (‘is bearer of’ some ‘contact’)) or (‘has occurrent part’ some ‘multi-organism behavior’ and (‘has participant’ min 2 ‘organism’)))

**Fig. 1:**
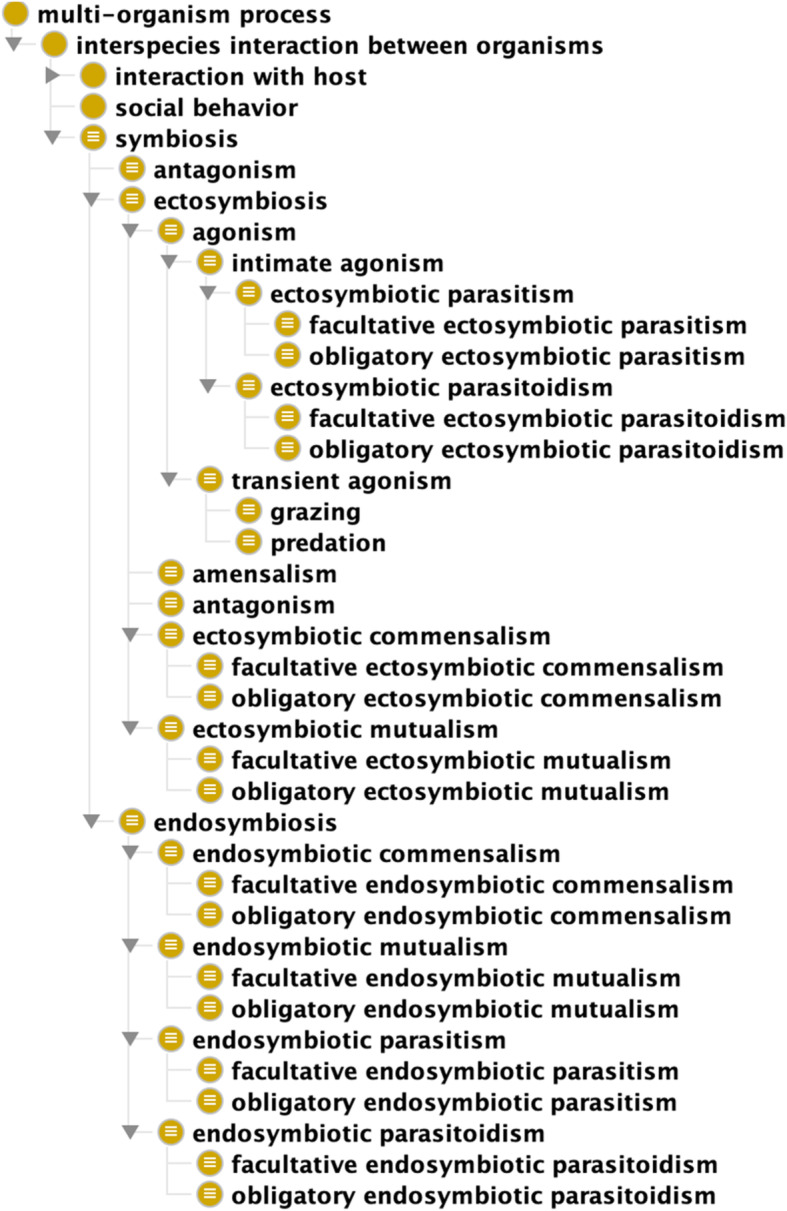
Class hierarchy of ‘symbiosis’

We assigned this class as a subclass of *interspecies interaction between organisms* (GO:0044419) (itself a subclass of *multi-organism process* (GO:0051704)), which we imported from GO (Fig. 1). GO defines *interspecies interaction between organisms* (GO:0044419) as *“Any process in which an organism has an effect on an organism of a different species.”* Thus, through subsumption we fulfilled the first and second aforementioned criteria that we established for defining ‘symbiosis’, which are that a symbiotic interaction is a type of process and that this process must occur among individual organisms of two or more different species.

We fulfill the third criterion by explicitly stating that some degree of intimacy must exist between the interacting organisms. Here, we use “intimate association” to mean that the interacting organisms are either in physical contact with one another or with another’s biological products or are engaged in some form of multi-organism behavior, the latter of which is defined in GO as *“Any process in which an organism has a behavioral effect on another organism of the same or different species”* (GO:0051705), although our definition of symbiosis would restrict this behavioral interaction to being solely interspecies. Finally, we also specify that the number of organisms of different species participating in a symbiotic interaction does not need to be restricted to two since there are several examples in nature of tripartite interactions (i.e., symbiotic interactions that involve three organisms of separate species. For example, it is now known that many lichens are a composite of organisms from three species that are engaged in a symbiosis with each other).

Because our overall goal was a comprehensive representation of symbiotic interactions in Apollo-SV, we compiled a set of candidate interaction types (Table 1) and a set of characteristics with which to define them based on available evidence in the literature [5]. The characteristics that differentiate subtypes of symbiosis that we identified are:

1. The outcome of the interaction for each organism (i.e., harm vs. benefit to the organism that results from the symbiosis)
2. The locations of the organisms relative to one another throughout the interaction
3. Whether the interaction is necessary for—or merely advantageous to—fulfilling one or more biological functions of one or more of the organisms

**Table 1:** Types of interactions considered for representation

Interaction	Outcomes	Level of Intimacy
mutualism	+/+	Varies
commensalism	+/0	Varies
agonism	+/−	Varies
amensalism	−/0	Varies
antagonism	−/−	Varies
parasitism	+/−	High
parasitoidism	+/−	High
predation	+/−	Low
grazing	+/−	Low

Legend: ‘+’ = benefit; ‘-‘= harm; ‘0’ = neutral

More specifically, the outcome of the interaction for each organism is defined as being is beneficial, harmful, or neutral (that is, neither harmful nor beneficial). Thus, in symbiotic interactions between two organisms, we can say that both were benefitted by the interaction; both were harmed; one was benefitted, while the other was harmed; and so on. One exception to these interactions is an interaction in which neither organism is affected—often referred to as “neutralism”. The scientific community generally considers “neutralism” to be non-existent. Therefore, we excluded the term ‘neutralism’ and this combination of outcomes from further consideration and analysis.

With respect to relative locations of organisms with respect to each other, we distinguish between interactions in which one organism is inside of another (i.e., endosymbiotic interactions) and those in which all participants remain physically external to one another (i.e., ectosymbiotic interactions). This distinction is necessary for determining the level of intimacy of interactions, as there is a high level of intimacy in endosymbiotic interactions, whereas in ectosymbiotic interactions the level of intimacy is lower. From our analysis, endosymbiotic interactions only occur in a subset of the interactions listed in Table 1 (i.e., mutualism, commensalism, parasitism, and parasitoidism), whereas ectosymbiosis can occur in any of the types.

The third feature—necessity vs. advantage only—distinguishes between interactions that are necessary vs. those that are merely advantageous to at least one organism. Interactions that are necessary for the fulfilment of some biological function of an organism, such as those between various tick species and the host animals that they parasitize, are commonly referred to as “obligate” interactions. Note that obligate interactions, although typically thought of as being required for life itself, also encompass situations where the interaction is obligate for reproduction (but not life). On the other hand, interactions that are advantageous, but not necessary, for at least one interacting organism are referred to as “facultative.” We explain in the following sections the details about how we defined each criterion ontologically.

### Defining the characteristics of interactions

To represent each subtype of symbiotic interaction (Table 2), we utilized each of the aforementioned characteristics, which first required a rigorous definition for the classes associated with each. To formalize the outcomes of interactions, we created two new classes—*bodily harm* (APOLLO_SV:00000371) and *bodily benefit* (APOLLO_SV:00000372):

- *bodily harm = def. - A process consisting of a change in the structure integrity of some anatomical structure that weakens or strengthens some homeostasis disposition or function inhering in that anatomical structure, such that the strength of the disposition (function) deviates outside of the range that is necessary to maintain in homeostatic range those bodily qualities that its realization influences.*
- *bodily benefit = def. - A process that facilitates the realization of one or more normal homeostasis dispositions borne by an organism and/or one or more causally relatively isolated parts of the organism.*

**Table 2:** Types of Symbiotic Interactions

Term	Definition
agonism	An ectosymbiosis that results in harm to one of the participating organisms and benefit to another participating organism
intimate agonism	An agonism where the participating organisms, although physically external to one another, nevertheless have close and persistent physical contact for most or all of the interaction
ectosymbiotic parasitism	An intimate agonism in which the harm is not fatal to the host
ectosymbiotic parasitoidism	An intimate agonism in which the harm is fatal to the host
transient agonism	An agonism where the participating organisms maintain significant physical separation from one another through most or all of the interaction
grazing	A transient agonism in which the harm is not fatal
predation	A transient agonism in which the harm is fatal
amensalism	An ectosymbiosis that results in harm to one of the participating organisms but neither harms nor benefits the other participating organisms
antagonism	An ectosymbiosis that results in harm to all participating organisms
ectosymbiotic commensalism	An ectosymbiosis that results in benefit to one participating organism and neither harm nor benefit to the other participating organisms
ectosymbiotic mutualism	An ectosymbiosis that results in benefit to all participating organisms
endosymbiotic parasitism	An endosymbiosis that results in benefit to the parasite and harm to the host
endosymbiotic parasitoidism	An endosymbiosis that results in benefit to the parasitoid and death to the host
endosymbiotic commensalism	An endosymbiosis that results in benefit to one organism and neither harm nor benefit to the other participating organisms
endosymbiotic mutualism	An endosymbiosis that results in benefit to all participating organisms
obligatory ectosymbiotic parasitism	An ectosymbiotic parasitism that realizes a biological function that inheres in some proper part of the parasite
obligatory ectosymbiotic parasitoidism	An ectosymbiotic parasitoidism that realizes a biological function that inheres in some proper part of the parasitoid
obligatory ectosymbiotic commensalism	An ectosymbiotic commensalism that realizes a biological function that inheres in some proper part of the commensal organism that benefits
obligatory ectosymbiotic mutualism	An ectosymbiotic mutualism that realizes a biological function that inheres in some proper part of at least one of the organisms participating
obligatory endosymbiotic parasitism	An endosymbiotic parasitism that realizes a biological function that inheres in some proper part of the parasite
obligatory endosymbiotic parasitoidism	An endosymbiotic parasitoidism that realizes a biological function that inheres in some proper part of the parasitoid
obligatory endosymbiotic commensalism	An endosymbiotic commensalism that realizes a biological function that inheres in some proper part of the commensal organism that benefits
obligatory endosymbiotic mutualism	An endosymbiotic mutualism that realizes a biological function that inheres in some proper part of at least one of the organisms participating
facultative ectosymbiotic parasitism	An ectosymbiotic parasitism that realizes some facultative parasite role inhering in an organism that is also realized by a bodily benefit to that organism
facultative ectosymbiotic parasitoidism	An ectosymbiotic parasitoidism that realizes some facultative parasitoid role inhering in an organism that is also realized by a bodily benefit to that organism
facultative ectosymbiotic commensalism	An ectosymbiotic commensalism that realizes some facultative commensal role inhering in an organism that is also realized by a bodily benefit to that organism
facultative ectosymbiotic mutualism	An ectosymbiotic mutualism that realizes some facultative mutualist role inhering in an organism that is also realized by a bodily benefit to that organism
facultative endosymbiotic parasitism	An endosymbiotic parasitism that realizes some facultative parasite role inhering in an organism that is also realized by a bodily benefit to that organism
facultative endosymbiotic parasitoidism	An endosymbiotic parasitoidism that realizes some facultative parasitoid role inhering in an organism that is also realized by a bodily benefit to that organism
facultative endosymbiotic commensalism	An endosymbiotic commensalism that realizes some facultative commensal role inhering in an organism that is also realized by a bodily benefit to that organism
facultative endosymbiotic mutualism	An endosymbiotic mutualism that realizes some facultative mutualist role inhering in an organism that is also realized by a bodily benefit to that organism

Here, we make use of the class *homeostasis* (OGMS:0000032) from the Ontology for General Medical Science (OGMS) to differentiate between processes that result in a departure from an organism’s normal physiological state vs. those processes that promote the normal state [28]. At the time of this writing, this class is undefined in in the OWL implementation of OGMS. However, prior work by Scheuermann et al. [29] on representing diseases and diagnoses in OGMS defined ‘homeostasis’ as *“[A] disposition of the whole organism (or of some causally relatively isolated part of the organism, such as a single cell) to regulate its bodily processes in such a way as (1) to maintain bodily qualities within a certain range or profile and (2) to respond successfully to departures from this range caused by internal influences or environmental influences such as poisoning.”* In the Basic Formal Ontology (BFO), dispositions are treated as realizable entities that exist in material entities by virtue of the physical makeup of those entities and are only realized when certain processes occur [30]. Thus, a homeostasis disposition is realized only under certain physiological conditions in which bodily qualities of the organism move outside some normal range. A failure to return one or more bodily qualities to a range that is within what is normal is therefore indicative of some previous harm to the organism. On the other hand, any process that helps to correct or improve an organism’s ability to regulate its bodily qualities to within their normal ranges is beneficial to the organism. Although the work by Scheuermann et al. was originally intended for the context of clinical medicine, we find it to be generalizable to symbiotic interactions since the processes of harm and benefit that occur as a result of such interactions overlap with those that are observed in clinical settings.

The relative location of interacting organisms to each other poses an additional challenge. Namely, the issue here is about whether cavities or spaces within the body (e.g., the space enclosed by the inner lining of the gastrointestinal tract) are parts of the body. To address this, we refer to work in OGMS defining *extended organism* (OGMS:0000087): *“An object aggregate consisting of an organism and all material entities located within the organism, overlapping the organism, or occupying sites formed in part by the organism.”* We therefore consider such spaces to be part of the body, and as a consequence, any organisms that dwell within such spaces to be located within the larger host organism. With this in mind, we propose the following definitions and equivalent class axioms for *ectosymbiosis* (APOLLO_SV:00000337) and *endosymbiosis* (APOLLO_SV:00000354):

- *ectosymbiosis = def. - A symbiosis where the organisms that are interacting remain physically external to one another.*
- ‘symbiosis’ and (‘has participant’ some (‘organism’ and (‘spatially disjoint from’ some ‘organism’)))
- *endosymbiosis = def. - A symbiosis where one organism is physically contained within another extended organism.*
- ‘symbiosis’ and (‘has participant’ some (‘organism’ and (‘located in’ some ‘organism’)))

To distinguish facultative vs. obligatory symbioses, we used BFO’s *role* (BFO:0000023) and *function* (BFO:0000034) classes, respectively. In BFO, roles are defined as realizable entities that exist in a bearer due to one or more external circumstances that the bearer does not have to be in [31]. Moreover, unlike dispositions, an independent continuant can cease to be the bearer of a role without undergoing any change to its physical makeup. We make use of this distinction to define facultative interactions since these interactions can be thought of as “optional” for facultative organisms in the sense that the interaction is only advantageous for the overall fitness of the organism, as opposed to being necessary for its survival and/or reproduction. For example, the so-called “brain-eating amoeba,” *Naegleria fowleri* (taxid 5763), typically feeds on bacteria found in freshwater bodies. However, when the opportunity avails itself, it will often infect humans. This transition from preying on bacteria to becoming a human pathogen does not arise as a result of some physiological change in the amoeba, but rather is due to it coming in contact with a human host. As such, we define facultative interactions as *“An* x *interaction that realizes some facultative role that inheres in an organism and is realized by a bodily benefit to that organism,”* where ‘x’ represents some type of symbiotic interaction (e.g., mutualism, parasitism) and where a facultative role is a role that inheres in an organism that, when realized, facilitates some sort of bodily benefit to the organism that could otherwise have been realized independent of any symbiotic interaction.

This is in contrast to functions, which in BFO are defined as: “A function is a disposition that exists in virtue of the bearer’s physical make-up and this physical make-up is something the bearer possesses because it came into being, either through evolution (in the case of natural biological entities) or through intentional design (in the case of artifacts), in order to realize processes of a certain sort” [32]. Examples of this include the function of your heart to pump blood and the function of your neurons to transmit, receive, and regulate signals throughout the central nervous system. Because certain organisms depend on obligatory interactions to realize functions which are essential to survival or reproduction, we say that these interactions realize some biological function that inheres in that organism. Thus, our definition of obligatory symbiotic interactions takes the form of: *“An* x *interaction that exclusively realizes some biological function that inheres in some proper part of an organism,”* where ‘x’ refers to some type of symbiotic interaction. We say “exclusively realizes” because the function cannot be realized through other means.

### Referent tracking scenario 1: Ectoparasitism

In this scenario, we represent an ectoparasitic interaction (IUI-4) between a human host, Mr. Jones (IUI-1), and a colony of *T. rubrum* (taxid 1280) (IUI-2) (Tables 3 and 4). We assume that the skin of the posterior part of the right elbow of Mr. Jones (IUI-3) is part of Mr. Jones and therefore has existed throughout the same time interval that he has (t_1_), and that this particular anatomical region is where the ectoparasitic interaction is occurring. We can easily capture all instantiations and relationships, including the participation of both Mr. Jones and the colony of *T. rubrum* (taxid 1280) in the ectoparasitism.

**Table 3:** Entities and their Instantiations for Scenario 1

IUI	Entity	Temporal region	Type
IUI-1	Mr. Jones	t_1_	human being
IUI-2	Colony of *T. rubrum*	t_2_	object aggregate
IUI-3	Mr. Jones’ right elbow	t_1_	skin of posterior part of right elbow
IUI-4	Ectoparasitic interaction between the *T. rubrum* colony and Mr. Jones	t_3_	ectoparasitism

**Table 4:** Relationships among particulars in Scenario 1

IUI	Relation	IUI	Temporal region when relation holds	Notes
IUI-4	has-participant	IUI-1	t_3_	Ectoparasitism has participant Mr. Jones
IUI-4	has-participant	IUI-2	t_3_	Ectoparasitism has participant the colony of *T. rubrum*
IUI-4	occurs-in	IUI-3	t_3_	The ectoparasitism occurs in the skin of the posterior part of Mr. Jones’ right elbow
IUI-3	part-of	IUI-1	t_1_	The skin of the posterior part of Mr. Jones’ right elbow is part of Mr. Jones
t_3_	part-of-occurrent	t_1_	t_max_	The temporal interval occupied by the ectoparasitism is part of the temporal interval occupied by Mr. Jones existence

### Referent tracking scenario 2: obligatory Endocommensalism and obligatory Endoparasitism

This scenario represents multiple obligatory parasitic interactions between human hosts and the *P. vivax* malaria parasite (taxid 5855), as well as multiple obligatory commensal interactions between the *P. vivax* malaria parasite (taxid 5855) and an *A. gambiae* mosquito (taxid 7165) host (Tables 5 and 6). The main difference between these types of interactions, in terms of organism outcomes, is that the former interaction ends up harming the human host, while the latter has no effect on the mosquito host. Therefore, in this scenario, we represent an infected human host, Mr. Joaquin (IUI-4), who is the source of the *P. vivax* (taxid 5855) transmission, a mosquito vector (IUI-5) that acquires an aggregate of *P. vivax* parasites (taxid 5855) (IUI-6) when it bites Mr. Joaquin, the aggregate of *P. vivax* sporozoites (taxid 5855) (IUI-8) that are the offspring of the *P. vivax* parasites (taxid 5855), and Mrs. Chang (IUI-7) who is eventually infected by those sporozoites. We further assert that the original endoparasitic interaction (IUI-9) between the *P. vivax* malaria parasites (taxid 5855) (IUI-6) and Mr. Joaquin (IUI-4) occurred during the temporal interval denoted by t_9_. The temporal interval t_10_ is occupied by the obligatory endocommensal interaction (IUI-10) between the *P. vivax* malaria parasites (taxid 5855) (IUI-6) and the mosquito (IUI-5) that bit Mr. Joaquin (IUI-4). During the course of this interaction, *P. vivax* sporozoites (taxid 5855) (IUI-8) grow and develop in the mosquito (IUI-5) through another obligatory endocommensal interaction (IUI-12), which occurs during t_12_. Eventually, the sporozoites (IUI-8) are transmitted to Mrs. Chang (IUI-7) and a second obligatory endoparasitic interaction (IUI-11) unfolds during t_11_.

**Table 5:** Entities for Scenario 2

IUI	Entity	Temporal region	Type
IUI-4	Mr. Joaquin, the initial infected human	t_4_	human being
IUI-5	The particular *A. Gambiae* mosquito that bites Mr. Joaqin and Mrs. Chang	t_5_	*Anopheles gambiae*
IUI-6	Aggregate of *P. vivax* parasites that were transmitted from Mr. Joaquin to the *A. Gambiae* mosquito denoted by IUI-8	t_6_	object aggregate
IUI-7	Mrs. Chang, who was uninfected prior to coming in contact with the mosquito denoted by IUI-8	t_7_	human being
IUI-8	Aggregate of *P. vivax* sporozoites that developed in the mosquito denoted by IUI-8 and that infected Mrs. Chang	t_8_	object aggregate
IUI-9	Obligatory endoparasitic interaction between IUI-6 and IUI-4	t_9_	obligatory endoparasitism
IUI-10	Obligatory endocommensal interaction between IUI-6 and IUI-5	t_10_	obligatory endocommensalism
IUI-11	Obligatory endoparasitic interaction between IUI-8 and IUI-7	t_11_	obligatory endoparasitism
IUI-12	Obligatory endocommensal interaction between IUI-8 and IUI-5	t_12_	obligatory endocommensalism

**Table 6:** Relationships among particulars in Scenario 2

IUI	Relation	IUI	Temporal region when relation holds	Notes
IUI-9	has-participant	IUI-4	t_9_	The obligatory endoparasitism denoted by IUI-9 has participant Mr. Joaquin
IUI-9	has-participant	IUI-6	t_9_	The obligatory endoparasitism denoted by IUI-9 has participant the aggregate of *P. vivax* parasites denoted by IUI-6
IUI-10	has-participant	IUI-5	t_10_	The obligatory endocommensalism denoted by IUI-10 has participant the *A. Gambiae* mosquito denoted by IUI-5
IUI-10	has-participant	IUI-6	t_10_	The obligatory endocommensalism denoted by IUI-10 has participant the aggregate of *P. vivax* parasites denoted by IUI-6
IUI-11	has-participant	IUI-7	t_11_	The obligatory endoparasitism denoted by IUI-11 has participant Mrs. Chang
IUI-11	has-participant	IUI-8	t_11_	The obligatory endoparasitism denoted by IUI-11 has participant the aggregate of *P. vivax* sporozoites denoted by IUI-8
IUI-12	has-participant	IUI-5	t_12_	The obligatory endocommensalism denoted by IUI-12 has participant the *A. Gambiae* mosquito denoted by IUI-5
IUI-12	has-participant	IUI-8	t_12_	The obligatory endocommensalism denoted by IUI-12 has participant the aggregate of *P. vivax* sporozoites denoted by IUI-8
IUI-9	precedes	IUI-10	t_9_	The obligatory endoparasitism denoted by IUI-9 precedes the obligatory endocommensalism denoted by IUI-10
IUI-10	precedes	IUI-11	t_10_	The obligatory endocommensalism denoted by IUI-10 precedes the obligatory endoparasitism denoted by IUI-11
IUI-11	precedes	IUI-12	t_11_	The obligatory endoparasitism denoted by IUI-11 precedes the obligatory endocommensalism denoted by IUI-12
IUI-9	occupies-temporal-region	t_9_	t_max_	The obligatory endoparasitism denoted by IUI-9 occupies the temporal interval t_9_
IUI-10	occupies-temporal-region	t_10_	t_max_	The obligatory endocommensalism denoted by IUI-10 occupies the temporal interval t_10_
IUI-11	occupies-temporal-region	t_11_	t_max_	The obligatory endoparasitism denoted by IUI-11 occupies the temporal interval t_11_
IUI-12	occupies-temporal-region	t_12_	t_max_	The obligatory endocommensalism denoted by IUI-12 occupies the temporal interval t_12_
t_9_	part-of-occurrent	t_4_	t_max_	The temporal interval occupied by the first obligatory endoparasitism is part of the temporal interval in which Mr. Joaquin has lived
t_10_	part-of-occurrent	t_5_	t_max_	The temporal interval occupied by the first obligatory endocommensalism is part of the temporal interval in which the mosquito has lived
t_11_	part-of-occurrent	t_7_	t_max_	The temporal interval occupied by the second obligatory endoparasitism is part of the temporal interval in which Mrs. Chang has lived
t_12_	part-of-occurrent	t_5_	t_max_	The temporal interval occupied by the second obligatory endocommensalism is part of the temporal interval in which the mosquito has lived

### Referent tracking scenario 3: obligatory Endomutualism

In this scenario, we represented an obligatory endomutualistic interaction (IUI-18) between an *A. pisum* aphid (taxid 7029) (IUI-13) and a *B. aphidicola* bacterium (taxid 9) (IUI-14) (Tables 7 and 8). This interaction between the aphid and the *B. aphidicola* bacterium (taxid 9) is mutualistic because the bacterium produces several essential amino acids (e.g., tryptophan) for the aphid, which it does not obtain through its diet. Therefore, we assert for the interaction that the *B. aphidicola* bacterium (taxid 9) (IUI-14) is a participant in a tryptophan biosynthetic process (IUI-15) during time interval t_15_ that has as output some molecule of tryptophan (IUI-17). This same molecule of tryptophan (IUI-17) is then passed along to the aphid host (IUI-13) to be used as input to some process of translation (IUI-16), which occurs over the time interval t_16_. We then relate this back to the obligatory endomutualistic interaction (IUI-18) by asserting that the interval occupied by the tryptophan biosynthetic process (t_15_) and the interval occupied by the translation process (t_16_) each share a proper-part-of-occurrent relation with the interval occupied by the obligatory endomutualistic interaction (t_21_).

**Table 7:** Entities for Scenario 3

IUI	Entity	Temporal region	Type
IUI-13	The *A. pisum* aphid	t_13_	*Acyrthosiphon pisum*
IUI-14	A *B. aphidicola* bacterium	t_14_	Buchnera aphidicola (Acyrthosiphon pisum)
IUI-15	Tryptophan biosynthetic process in symbiont	t_15_	tryptophan biosynthetic process
IUI-16	Translation process in host	t_16_	translation
IUI-17	Tryptophan	t_17_	tryptophan
IUI-18	Obligatory endomutualistic interaction between the adult *A. Pisum* aphid and the *B. aphidicola* bacterium	t_18_	obligatory endomutualism

**Table 8:** Relationships among particulars in Scenario 3

IUI	Relation	IUI	Temporal region when relation holds	Notes
IUI-15	has-participant	IUI-14	t_15_	The tryptophan biosynthetic process has participant the *B. aphidicola* bacterium
IUI-15	has-output	IUI-17	t_15_	The tryptophan biosynthetic process has the tryptophan molecule
IUI-16	has-participant	IUI-13	t_16_	The translation process has participant the *A. pisum* aphid
IUI-16	has-input	IUI-17	t_16_	The translation process has input the tryptophan molecule
IUI-15	precedes	IUI-16	t_15_	The tryptophan biosynthetic process precedes the translation process in the host
IUI-15	proper-part-of-occurrent	IUI-18	t_15_	The tryptophan biosynthetic process is a proper occurrent part of the obligatory endomutualism
IUI-18	occupies-temporal-region	t_18_	t_max_	The obligatory endomutualism occupies the temporal interval t_18_
t_15_	proper-part-of-occurrent	t_14_	t_max_	The temporal interval occupied by the tryptophan biosynthetic process is a proper occurrent part of the temporal interval in which the *B. aphidicola* bacterium has lived
t_16_	proper-part-of-occurrent	t_13_	t_max_	The temporal interval occupied by the translation process is a proper occurrent part of the temporal interval in which the aphid has lived
t_16_	proper-part-of-occurrent	t_18_	t_max_	The temporal interval occupied by the translation process is a proper occurrent part of the temporal interval occupied by the obligatory endomutualism
t_15_	proper-part-of-occurrent	t_18_	t_max_	The temporal interval occupied by the tryptophan biosynthetic process is a proper occurrent part of the temporal interval occupied by the obligatory endomutualism

## Discussion

Due to our need to represent pathogen-host interactions and the epidemics that arise from them, we expanded Apollo-SV to include classes for symbiotic interactions. To do so, we leveraged a realism-based approach to ontology development to create a total of 35 classes that represent symbiosis and its currently scientifically recognized subtypes. We also sought to re-use classes from other ontologies wherever possible. However, this process only resulted in the reuse of three classes due to several persistent and major difficiencies in existing representations of symbiotic interactions. One of the most egregious errors that we found in definitions for ‘symbiosis’ was circularity. Another issue that we encountered was definitions that were too restrictive on the types of interactions that symbiosis encompasses. Although it might partly be explained by the historic inconsistency in how ‘symbiosis’ has been defined, there were some definitions that did not mirror any of the alternative definitions that biologists and ecologists have used. For example, MeSH, for reasons unknown, excluded parasitism from being a type of symbiotic interaction, although it did include commensalism in addition to mutualism.

The third issue that we came across in current representations of symbiotic interactions was that the interactions are defined at the species level, rather than at the level of the individual organisms. This brings about an interesting question, however, about which of the two is more ideal. After all, when we think of organisms that are mutualists or parasites, we often invoke phrases and terms that imply the level of the species (e.g., when we say that deer ticks [taxid 6945] are parasites). Nevertheless, we believe that it is ontologically incorrect to define symbiotic interactions at the species level since the intance-level symbiotic interactions that humans observe or participate in in our day-to-day lives occur between individual organisms, rather than between entire species. Moreover, there is plenty of evidence of some fluidity in the outcomes of a symbiotic interaction for each participating organism over time. By this, we mean that any given instance of an interspecies interaction may not instantiate only one type of symbiotic interaction at every time point throughout the interaction. For example, *Staphylocauccus aureus* bacteria (taxid 1280) that exist on the skin and upper respiratory tracts of some humans will likely maintain a commensal interaction with their human host for most or all of their life course. However, under certain conditions, this commensal interaction might end and a parasitic interaction (that is, infection) subsequently begin if the *S. aureus* (taxid 1280) colonies become pathogenic. This does not mean, though, that every individual that is a member of the species *S. aureus* (taxid 1280) becomes pathogenic, just as it does not mean that every member of *S. aureus* (taxid 1280) is commensal (or even live on or within a host at all).

Our use of a referent tracking based analysis to represent individual-level data about symbiotic interactions demonstrates the generalizability of this paradigm to disciplines other than the ones our scenarios directly addressed, as information about symbiotic interactions is directly relevant to numerous fields, such as zoology, mycology, botany, ecology, epidemiology, and microbiology. However, any discipline that would benefit from the ability to reason over instance-level data would especially be likely to benefit from this approach. In addition, our referent tracking analysis helped shed more light on the issue of whether to define symbiotic interactions at the species level or the individual level. Particularly, in Scenario 2, the *P. vivax* parasite (taxid 5855) is at times engaged in an endoparasitic interaction with a human host, while at other times it is engaged in an endocommensal interaction with a mosquito host. Furthermore, scientific data from which species-level inferences are made are recorded at the level of observations of individuals, and thus encoding of data with ontologies requires representations of individual-level—not species-level—interactions. As such, we chose pragmatically to define symbiotic interactions at the level of the individual organism.

It is important to note that the historic debate about terminological consistency in the definition of ‘symbiosis’ has largely been waged between two camps: one that favors the de Bary definition, which encompasses mutualism, parasitism, and commensalism, and also considers intimacy to be a requirement of such interactions; and another that equates symbiosis with mutualism. Although we have stated our preference for the de Bary definition, which also happens to be shared by a growing supermajority of biologists and ecologists, we are aware that our work here proposes the addition of several other subclasses of ‘symbiosis’ outside of the traditional ‘mutualism’, ‘parasitism’, and ‘commensalism’ distinctions. For example, while de Bary never considered predation to be a type of symbiosis, we have included it in the term hierarchy of ‘symbiosis’. Our decision to do so hinges on the fact that many of these terms bear a close resemblance to one of the three original types of symbiotic interactions, as noted by Martin and Schwab [5], and that all of them possess each of the characteristics that we identified of symbiotic interactions. Returning to the aforementioned example of predation, we note that parallels between predation and parasitism are frequently discussed in biology and ecology textbooks, with some even suggesting that parasitism is a subtype of predation (certainly, such parallels are even more apparent for parasitism and parasitoidism) [5]. Although we do not agree with such a classification of parasitism as predation, we acknowledge that the ontological nature of such interactions (i.e., the close intimacy that exists between a predator and its prey throughout their interaction, in addition to the positive/negative outcomes for predator and prey, respectively) overlaps significantly. However, we take the endosymbiosis vs. ectosymbiosis distinction as being ontologically more important than the degree of intimacy and the outcomes of the interaction. If one were to take either degree of intimacy or outcomes as the primary ontological distinction, then one would end up with a taxonomy that places predation and parasitism as siblings at a minium. And taking it one step further, if one then defined predation as being equivalent to a symbiosis with a high level of intimacy where one organism benefits and one organism is harmed (regardless of ectosymbiosis vs. endosymbiosis), then indeed one would place parasitism as a subtype of predation (although one would then need a new term for parasitism’s sibling, something akin to ‘ectosymbiotic predation’). Similar considerations also apply to other subtypes of symbiosis for which we created classes (i.e., parasitoidism, grazing, antagonism, amensalism, and agonism). We argue that our classification of each of these types of interactions as being symbioses follows in the spirit of Anton de Bary’s original definition of “the living together of unlike organisms” and his further clarifications of what he meant.

We did encounter one term in the literature—‘neutralism’—that, according to all available evidence, does not seem to refer to any type of interspecies interaction that exists in reality, and we therefore did not define the term. This decision stems from the principles of ontological realism so described by Smith and Ceusters [16], which maintain that ontology classes must possess a one-to-one correspondence with universals in reality, which we know to exist through observation and study of the particulars that instantiate them. According to the usages of ‘neutralism’ that we encountered [5], the definition of the term can be summed up as “*A symbiosis in which none of the organisms are affected.”* Independent of the fact that ‘neutralism’ doesn’t appear to refer to any kind of interspecies interaction in reality, this term is problematic for another reason, which is that symbiotic interactions, by definition, are processes in which the participating organisms undergo change. To say that none of the organisms are changed by a process of change in which they are participating is nonsensical. It is a logical contradiction. This contradiction becomes even more evident when we interpose the definition of GO’s *interspecies interaction between organisms* (GO:0044419) within the aforementioned definition for ‘neutralism’:

*“[Any process in which an organism has an effect on an organism of a different species] in which none of the organisms are affected.”*

There are a few limitations to our work. The first limitation centers on the definition of symbiosis that we have adopted and is namely that there still exists a small degree of disagreement on how to define and use the term ‘symbiosis’ within the fields of biology and ecology. Nevertheless, the overwhelming majority of usage is as we have defined it here. Also, given that the definitions that we proposed here were formulated based on rigorous attention to the current popular usage of ‘symbiosis’ and related terms, we believe that our work provides a valuable framework for organizing and analyzing data about such interactions. Another limitation that we have not worked out yet is whether symbiotic interactions between one organism and a population of organisms from the same species should be considered a single, one-to-many interaction or an aggregate of one-to-one interactions (e.g., the interaction(s) between a human host and its gut bacteria). Although our definition of ‘symbiosis’ does allow for interactions between more than two organisms, which would accommodate a one-to-many interaction between a host and a population of microorganisms, there may be reason to define it as multiple one-to-many interactions (e.g., if part of the population becomes pathogenic, while another part remains commensal or mutualistic). Note that in our RT analysis, for the sake of convenience we took the former approach: one interaction in which an entire colony or aggregate of sporozoites participates. Another limitation is in the vagueness of the phrase “close intimacy between organisms,” which we acknowledge can be interpreted or defined in multiple ways. Our initial inclination is that ‘intimacy’ refers to the degree and duration of physical or social contact between two organisms, such that at least one of the organisms is capable of directly producing a bodily benefit or harm in the other. Others may choose to include indirect, yet nonetheless close, contact between organisms in their definition of close intimacy, for example, when two organisms compete for the same resource. Whether restricting it to direct contact is preferential over the above alternative is something that may need to be addressed in future work. There also exists one limitation with the axiomatic definitions that we created for each class, which is that the lack of variables in description logics prevents us from, in case of a parasitic interaction, for example, saying that the organism that is harmed is different from the organism that is benefited. It also prevents us from saying that the bodily harm or benefit are part of the overall interaction process. Finally, although our proposed definition for ‘symbiosis’ accounts for interactions in which there are more than two participants of different species (e.g., tripartite interactions), there still remains work to be done on how to represent such interactions for each type of symbiotic interaction. For example, if all three organisms of different species benefit from the interaction, we can easily classify it as mutualism. However, if one organism benefits, the second organism is harmed and is the host, and the third organism is unaffected, then do we classify the interaction as commensalism, parasitism, or both? According to our current definitions, we would indeed classify such an interaction as being both a commensalism and a parasitism.

## Conclusions

We represented symbiotic interactions using a realism-based approach to ontology development. We reviewed the representation of symbiotic interactions in various terminologies, ontologies, and thesaurii and found them to be inadequate, largely because the definitions are either circular or too restrictive. We identified numerous subtypes of symbiotic interactions from the literature and defined them according to three characteristics: the outcome for each organism, the relative location of each organism to the other(s), and whether the interaction is facultative or obligatory. To this end, we made use of prior ontology work related to homeostasis and extended organisms. In total, we imported three classes from other ontologies and created 35 new classes in Apollo-SV. Given the large impact that symbiotic interactions have on human health, we believe that this work is an important step toward the development of computational tools for capturing and analyzing data about such interactions.

## Data Availability

The Apollo-SV ontology is available on GitHub at https://github.com/ApolloDev/apollo-sv/.

## Abbreviations

Apollo-SV: Apollo Structured Vocabulary
BFO: Basic Formal Ontology
CID: Concept identifier
CRISP: Computer Retrieval of Information on Scientific Projects
GO: Gene Ontology
IDO: Infectious Disease Ontology
IUI: Instance unique identifier
MeSH: Medical Subject Headings
MIREOT: Minimum Information to Reference an External Ontology Term
NIH: National Institutes of Health
NLM: National Library of Medicine
OBO: Open Biomedical Ontologies
OGMS: Ontology for General Medical Science
OWL2: Web Ontology Language version 2
RePORTER: Research Portfolio Online Reporting Tools
SNOMED CT: Systematized Nomenclature of Medicine—Clinical Terms
taxid: NCBI taxon identifier
